# Following the Decomposition of Hydrogen Peroxide in On-Site Mixture Explosives: Study of the Effect of the Auxiliary Oxidising Agent and Binder

**DOI:** 10.3390/molecules28165957

**Published:** 2023-08-08

**Authors:** Magdalena Fabin, Agnieszka Stolarczyk, Roman Zakusylo, Tomasz Jarosz

**Affiliations:** 1 Department of Physical Chemistry and Technology of Polymers, Silesian University of Technology, 44-100 Gliwice, Poland; 2 Shostka Institute, Sumy State University, 41100 Shostka, Ukraine

**Keywords:** hydrogen peroxide, explosive, Raman spectroscopy, decomposition

## Abstract

The issues of safety and its impact on both human health and the environment are on-going challenges in the field of explosives (EXs). Consequently, environmentally-friendly EXs have attracted significant interest. Our previous work, dedicated to on-site mixed (OSM) EXs utilising concentrated hydrogen peroxide (HTP) as an oxidising agent, revealed that the gradual decomposition of HTP may be harnessed as an additional safety measure, e.g., protection from theft. The rate of HTP decomposition is dependent on the OSM components, but this dependence is not straightforward. Relevant information about the decomposition of HTP in such complex mixtures is unavailable in literature. Consequently, in this work, we present a more detailed picture of the factors influencing the dynamics of HTP decomposition in EXformulations. The relevant measurement and validation methodology is laid out and the most relevant factors for determining the rate of HTP decomposition are highlighted. Among these, the choice of auxiliary oxidising agent is of particular relevance and it can be seen that the choice to use ammonium nitrate (AN), made in previous works dealing with HTP-based EXs, is sub-optimal in terms of maintaining the stability of HTP. Another important finding is that glass microspheres are not as inert to HTP as would be expected, as replacing them with polymer microspheres significantly slowed the decomposition of HTP in the investigated OSM samples.

## 1. Introduction

Explosives (EXs) are extensively used for civilian purposes, with blasting operations playing a crucial role in mining (underground and open pit) [1,2,3], civil engineering (demolitions, tunnel construction) [4,5] and materials processing (e.g., explosive welding) [6]. Virtually all explosives currently used for the above purposes contain ammonium nitrate, nitric acid esters or nitrocompounds. Despite their widespread use in blasting operations, such EXs are burdened by significant drawbacks, the most significant of which are the following:

- Manufacture from non-renewable resources via highly energy-intensive processes;
- Significant threat to human health and to the environment in the case of nitric acid esters and nitrocompounds [7,8,9,10,11];
- Emission of large amounts of toxic and highly corrosive gases (carbon monoxide, nitrogen oxides) upon detonation [12];
- Gradual decomposition, particularly in the case of liquid nitric acid esters, as well as gradual leakage of liquid nitroesters from the explosives, necessitating the use of stabilising and anti-leakage agents;
- Susceptibility to misfires (primarily in the case of explosives based on ammonium nitrate)—currently one of the primary threats associated with blasting operations [13,14];
- Susceptibility to theft and subsequent criminal misuse, due to maintaining the ability to detonate even following improper storage or a misfire during blasting [15];
- Significant risk of fire/explosion during production, transport and disposal, due to susceptibility to undergo detonation caused by various stimuli (e.g., impact, friction) [16,17,18];

The above issues have long been recognised in the field and safety considerations are likely the key reason for the development of emulsion explosives [19], which contain no nitric acid esters or nitrocompounds, while achieving only slightly lower energetic parameters than explosives utilising such compounds. Although bulk emulsion explosives partially resolve some of the above issues, they are not a satisfactory solution, as their energetic performance is relatively weaker than that of both traditional explosives and of cartridged emulsion explosives. Moreover, cases of theft and criminal misuse of emulsion explosives have been reported [15]. Consequently, the resolution of the entire set of these issues necessitates a different material solution.

One potential avenue of research is based on the use of concentrated (>50 wt.%) hydrogen peroxide (HTP) solutions, as they contain no nitrogen compounds, while being capable of undergoing detonation, with a velocity of detonation (VoD) of 6.6 km/s being reported [20]. By itself, however, HTP solutions have a large critical diameter (40.6 mm at 50 °C and decreasing with increasing temperature), making them impractical for any blasting operations. Consequently, a variety of fuels, both organic and inorganic, have been tested in HTP-based EX formulations [21], including the study of raw working solutions from the HTP manufacturing process

More recently, mixtures of HTP with components of the working solutions used to produce HTP on an industrial scale via the anthraquinone process were found to be explosive and sensitive to initiation by impact in a broad range of chemical concentration ratios [22]. Relatively little information is available on the energetic performance of such liquid explosive mixtures, likely due both to the high reactivity of HTP and to the generally marginal application of liquid explosives in blasting operations. It is, however, worth noting that HTP has been used as an auxiliary source of energy in studies on the electrical explosion of wire arrays [23]. Studies on peroxyhydrates and their ability to undergo detonation have also been reported [24], but such compounds, by themselves, show limited performance, with an infinite diameter detonation velocity of less than 4 km/s and 40% TNT equivalence in terms of air blast being reported.

Due to the above, more recent works on HTP-based explosives largely include gelling agents in their formulations, granting the materials minor mechanical strength and resistance to spillage while limiting the contact of HTP with potential contaminants [25]. This modification of HTP-based explosives has attracted greater research interest and such explosives were labelled as “green” explosives [26], primarily due to the fact that their formulations do not involve any nitrogen compounds and, hence, cannot lead to nitrogen oxide emissions upon detonation [27].

In terms of energetic parameters, HTP-based explosives have exhibited moderately high performance, varying with the specific choice of fuel, gelling agent and auxiliary compounds, as well as with explosive charge dimensions and casing (Table 1). In general, however, performance comparable with that of nitroester-based explosives (e.g., dynamites) and exceeding that of ammonium nitrate-based explosives was observed, while good initiation reliability was reported, possibly indicating that these materials are not as prone to misfires as explosives based on ammonium nitrate. It should also be noted that such materials can be produced on-site from non-explosive components, much as in the case of bulk emulsion explosives, mitigating another of the abovementioned drawbacks of traditional explosives.

One potential challenge in the practical application of HTP-based explosives stems from the decomposition of HTP, which can be spontaneous, caused by interactions of HTP with the components of the EX formulation or caused by interaction of HTP with the minerals constituting the walls of the boreholes. The first has been discussed above, the second is investigated experimentally in this work and the third is a consideration for future investigations. The nature of this third aspect is also highly dependent on the rock, in which blasting operations are conducted. This ties in with the type of mineral resources to be excavated, as e.g., coal deposits are often accompanied by sulfides, phosphates, chlorides, carbonates, sulfates and oxides [31]. Copper deposits, which are commonly excavated using explosives, offer a similar challenge, as they frequently co-exist with high amounts of sulfide minerals [32]. Many such minerals can act as catalysts for the decomposition of HTP and a strategy for mitigating their influence on HTP needs to be developed when considering the application of OSM-type explosives.

The issue of decomposition of concentrated hydrogen peroxide has been investigated to some extent in the literature [33], with the concentration of high-purity 90 wt.% H_2_O_2_ solutions decaying by approx. 1 wt.% per year when stored at 30 °C, but accelerating greatly with increased storage temperature (2 wt.% per 24 h when stored at 100 °C). It should be noted that the presence of contaminants can, but does not necessarily, expedite this decay, as seen by the fact that H_2_O_2_ solutions supplemented with 10 mg/dm^3^ of aluminium and 10 μg/dm^3^ of copper have been found to lose 2 wt.% and 24 wt.%, respectively, after 24 h of storage at 100 °C.

In the case of the OSM EXs, the other components of the formulations can act as “contaminants” and promote the decomposition of hydrogen peroxide. However, little information is available in the literature about the reactivity of any of these compounds with hydrogen peroxide. In terms of the reactivity of HTP with glycerine, literature reports that the reaction between the two substances takes place only in the presence of catalysts [34,35]. The impact of the other components of OSM formulations on the decomposition of HTP has not been investigated in literature. The lack of such fundamental information significantly impairs the design of HTP-based EXs, such as the investigated OSM formulations.

In our previous work, we investigated the impact of the addition of varied amounts and types of Al powders on both the energetic performance of the OSM EXs and on the decomposition dynamics of HTP used in their formulations [30]. That work was the first study to report on the decomposition dynamics of HTP in an EX formulation. The unexpectedly significantinfluence of even the microstructure of Al (i.e., flaked vs. atomised) on HTP decomposition has shown the need for a more in-depth investigation of the factors influencing this process.

Consequently, in this work, we have studied the changes in hydrogen peroxide concentrations over time in a series of OSM EX formulations, utilising several auxiliary oxidising agents (two grades of ammonium nitrate, potassium nitrate, sodium nitrate and calcium nitrate) and binders (guar gum, locust bean gum, hydroxyethylcellulose and gum arabic). The main intent behind this choice was to provide at least an initial insight into how some of the common components used in designing EX formulations affect the decomposition dynamics of HTP, as no relevant data appears to exist in current literature. Unlike the previous work, we have not included aluminium in the formulations of the tested explosives, so as to better focus on the more fundamental components. As such, the investigated OSM samples can be likened to variations on the OSM-0 formulation described in that previous work. The key improvement contained in this work, in relation to the state of the art, is that the impacts of each component in OSM formulations are compared and at least an initial explanation of the observed effects is proposed. This information constitutes the foundation for better integrating the principles of chemical sciences into the design process of EXs containing HTP.

## 2. Results and Discussion

### 2.1. OSM Density Investigation

**Figure 1:**
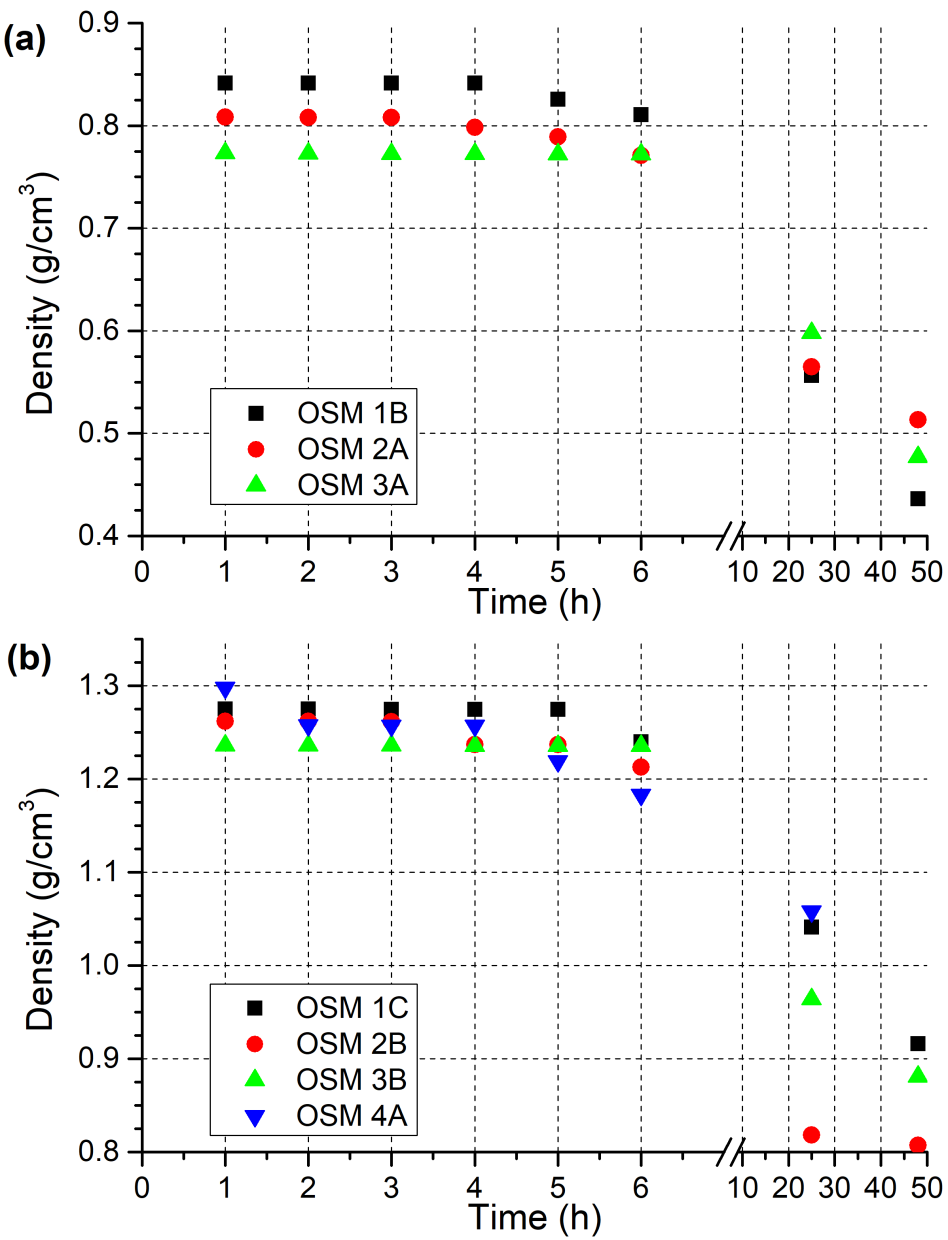
Changes in the density of OSM samples containing (**a**) polymer microspheres (PM); (**b**) glass microspheres (MS) over time.

The significance of the choice of components in OSM-type explosives was well-evidenced by the comparison of even the initial densities of the samples produced using PM and MS, even though only 0.95 wt.% of either component was used. The initial densities of the OSM formulations (Table 2) utilising PM (**1B**, **2A**, **3A**) were found to be on the order of 0.8 g/cm^3^, whereas formulations produced using MS (**1C**, **2B**, **3B**, **4A**) exhibited initial densities on the order of 1.2–1.3 g/cm^3^ (Figure 1).

In all cases, the passage of time resulted in a gradual decrease in OSM sample density. This stems from the fact that the decomposition of HTP in the OSM samples results in the release of gas bubbles (oxygen originating from spontaneous HTP decomposition, carbon dioxide originating from HTP reactions with the organic components of the formulations). The formation and presence of such bubbles in the bulk of the samples was observed during the measurements, much as in the case of our previous work. Although the choice of microsphere material shaped the initial density of the samples to a high extent, its effect on the changes of HTP concentration (and therefore density) over time was less pronounced, being comparable to that of the other components in the formulations.

Over the course of the first three hours of the experiments, the changes in OSM sample density were virtually negligible. In the following three hours, the decrease in density appeared to accelerate, even though the decomposition of HTP, shown by Raman spectroscopy, appeared to decelerate. This may stem from the fact that the initially forming gaseous byproducts of HTP decomposition are soluble to some extent in the OSM formulations and only after this solubility is exceeded do they evolve in the form of gas bubbles. After 24 h have elapsed, a significant decrease in density (on the order of 20–35% of the initial density) was observed for all samples, with the magnitude of this decrease being a function of both the microsphere material and choice of auxiliary oxidising agent.

### 2.2. Time-Resolved Raman Spectroscopy

**Figure 2:**
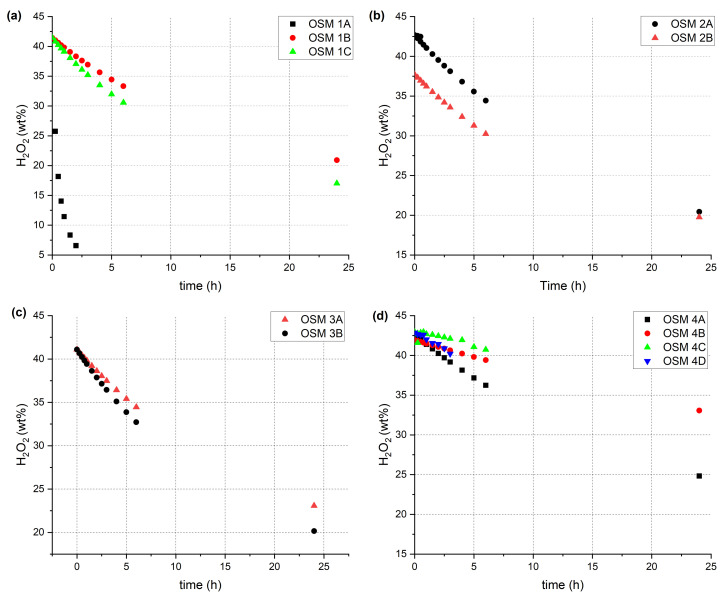
Changes in the effective concentration of hydrogen peroxide in OSM samples: (**a**) **1A**–**1C**; (**b**) **2A**–**2B**; (**c**) **3A**–**3B**; (**d**) **4A**–**4D**, over time.

**Figure 3:**
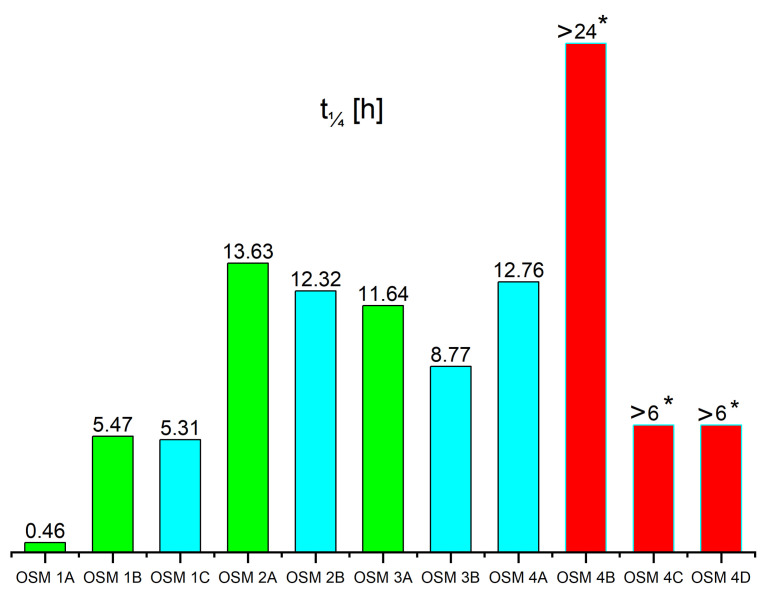
Comparison of the time required for the concentration of hydrogen peroxide in the OSM sample to decrease by to fourth of the initial measured concentration (t_1/4_). Samples containing polymer microspheres and glass microspheres have been colour-coded as green and teal, respectively. Samples in whose case the t_1/4_ exceeded the duration of the measurement are marked in red and labelled with an asterisk.

The dynamics of HTP decomposition in the investigated systems can be compared indirectly, by analysing the evolution of hydrogen peroxide concentration over time (Figure 2), or directly, based on the time required for the initially measured concentration of hydrogen peroxide to decrease by a fourth (Figure 3). This quarter-time was chosen instead of the more commonly employed reaction half-time, due to both the limited decay of hydrogen peroxide during the experiments and to avoid confusion surrounding a strictly kinetic parameter.

Our initial work [30] on OSM explosives relied on utilising commercially available ammonium nitrate (AN) as the auxiliary oxidising agent. In this work, we included the highly-porous ammonium nitrate prills (UltrAN) in our investigations for comparison (Figure 2a). Interestingly, the use of UltrAN (OSM **1A**) resulted in an extremely rapid decomposition of hydrogen peroxide (t_1/4_ of 0.46 h). Conversely, samples containing AN (OSM **1B** and **1C**) were found to show much higher stability (t_1/4_ of 5.47 and 5.31 h, respectively), containing in excess of 15 wt.% hydrogen peroxide after 24 h.

OSM explosives containing other auxiliary oxidising agents, i.e., SN (OSM **2A** and **2B**, (t_1/4_ of 13.63 and 12.32 h, respectively)), PN (OSM **3A** and **3B**, (t_1/4_ of 11.64 and 8.77 h respectively)) and CN (OSM **4A**–**4D**, (t_1/4_ of 12.76, >24, >6 and >6 h respectively)), underwent a notably slower decomposition than those containing ammonium nitrate. In the case of CN, more than 30 wt.% of hydrogen peroxide was retained within the explosive sample after 24 h, corresponding to a loss of less than a fourth of the initial amount of hydrogen peroxide.

The rapid decomposition of HTP in AN-bearing samples is in line with literature findings, as solutions composed of water, ammonia and hydrogen peroxide were found to have their concentrations of both ammonia and hydrogen peroxide decrease over time, even at room temperature and in the absence of hydrogen peroxide decomposition catalysts [36]. The reason for the faster observed decay of hydrogen peroxide in OSM samples containing ammonium nitrate (AN or UltrAN) than in the case of samples containing other auxiliary oxidising agents (SN, PN or CN) may be the fact that hydrogen peroxide is known to oxidise ammonia [37]. This reaction is promoted by light exposure, but takes place at a slower rate (due to lower concentrations of radicals produced from the homolytic breakage of the peroxide bond) in dark conditions. In our case, the other components of the OSM formulations may be sufficient promoters of the breakage of the peroxide bond, such that the effects of this reaction (i.e., faster hydrogen peroxide decay) may be observed. No such reaction has been observed for sodium, potassium or calcium cations, possibly explaining the difference in hydrogen peroxide decomposition dynamics. Interestingly, these results coincide only to some extent with the results of density measurements (e.g., the density of OSM **1C** being higher after 24 h than that of OSM **2B** and **3B**), indicating that the OSM samples may vary in terms of their ability to retain gaseous byproducts, whether by solubility or by entrapment of gas bubbles due to the viscosity of the samples.

Another important factor influencing the decomposition of hydrogen peroxide is the choice of the material constituting the microspheres. Comparison of hydrogen peroxide concentration changes over time for samples containing polymer microspheres (PM), i.e., OSM **1B**, **2A** and **3A**, with those containing glass microspheres (MS), i.e., OSM **1C**, **2B** and **3B** reveals that the use of PM results in slower hydrogen peroxide decomposition than if MS are used. This trend is observed regardless of the choice of auxiliary oxidising agent, although its magnitude varies to some extent. The use of PM typically results in hydrogen peroxide concentrations after 24 h being higher by approx. 2–5% than in the case of samples containing MS, which is readily apparent by comparison of the respective t_1/4_ values. The likely reasons for this trend are that PM show lesser wettability by hydrogen peroxide than MS and that breakage of MS results in porous fragments, on which hydrogen peroxide may be adsorbed, promoting its decomposition.

The issue of HTP decomposition in various containers has been investigated in literature [38], showing that HTP in glass containers decomposes rapidly, unless such a container is subjected to a passivation procedure. The proposed passivation procedure involves rinsing with hot nitric acid, distilled water and, finally, with HTP. As such, it is entirely inapplicable to the treatment of glass microspheres. Instead, polymer containers (e.g., polyethylene, polyfluorocarbons) are suggested as less reactive materials upon contact with HTP. This is in line with our comparison of the dynamics of hydrogen peroxide decay in OSM samples produced using MS and PM. However, no mechanism has been proposed to explain either the reactivity of HTP with glass or the lack of it in the case of polymer materials. Consequently, further research would be needed to present a detailed qualitative and quantitative description of this phenomenon, as it is impossible to exclude the presence of catalytic (in terms of hydrogen peroxide decomposition) impurities in the MS and their lack in PM.

The choice of utilised binder (OSM **4A**–**4D**) has a significant effect on both the dynamics of hydrogen peroxide decomposition of the OSM explosives and on their mechanical properties. Merely altering the structural features of the two investigated galactomannan polymers (GG and LBG), i.e., lowering the frequency of galactose side groups by 50%, results in an approx. 5% higher hydrogen peroxide concentration after 24 h (OSM **4B**). Transitioning to a different polysaccharide system (i.e., arabinose-based GA) or a modified polysaccharide (i.e., HEC) brings about significant changes, not only to hydrogen peroxide decomposition dynamics, but also to the observed consistency of the samples. Where OSM **1A**–**4B** can be described as viscous fluids, the HEC-bearing OSM **4C** exhibits sufficient viscosity to retain its shape even upon stirring. Conversely, the GA-bearing OSM **4D** shows low viscosity and rapidly undergoes phase separation.

It should be noted that the results of the conducted measurements show the existence of significant discrepancies between the composition of the OSM formulations that would be considered optimal in terms of (1) achieving the highest energetic properties; (2) exemplifying the best choice of components in line with typical explosive formulation design guidelines; (3) minimising the rate of hydrogen peroxide decomposition.

Considering the first of the above criteria, the density of the samples would be expected to have a direct effect on their velocity of detonation (VoD). In general, formulations containing glass microspheres exhibit noticeably higher density than the samples containing polymer microspheres. This difference in density is maintained over time and would be expected to translate to a comparable difference in VoD values for the formulations. The pressure of detonation would be a function of the amount of gaseous detonation products evolving from a unit mass of the OSM formulations, but this amount would vary only to a small extent based on the choice of the auxiliary oxidising agent, as small amounts of those agents are used in the formulations. The choice of the other components would have a negligible effect on this parameter, due to (1) the elemental composition of the investigated polysaccharide binders being virtually identical; (2) the microspheres comprising less than 1 wt.% of the formulations. Consequently, the OSM **4A** formulation, which exhibits the highest initial density, would likely have an optimal composition in terms of energetic properties.

In regards to the second criterion, the choice of any oxidising agent other than ammonium nitrate can be seen as sub-optimal, as, upon detonation, these oxidising agents yield solid ash that does not contribute to the performance of the OSM formulations. Again, formulations with higher densities would be preferred, so as to maximise the VoD of the explosive. Therefore, in this regard, OSM **1C** would be seen as having an optimal composition.

In terms of the last criterion, the experimental results presented in this work show that the decomposition of hydrogen peroxide was the slowest in the case of the OSM **4B** formulation. This formulation can be seen as only slightly inferior to OSM **4A** in terms of the first criterion, due to its lower density. Consequently, OSM **4B** can be considered as the most promising from among the investigated OSM formulations.

## 3. Materials and Methods

The reagents and chemicals used in this work are listed in Table 3.

### 3.1. Preparation of On-Site Mixture Samples

First, the auxiliary oxidising agent (UltrAN, AN, SN, PN or CN), binder (GG, LBG, HEC or GA), glycerine and the reference substance (MgSO_4_) were pre-mixed using a mechanical stirrer at 400 RPM for approx. 15 min. Next, the stirring rate was reduced to 100 RPM (to minimise MS breakage) and glass microspheres were added and mixed into the formulation for approx. 5 min. Lastly, HTP was added and mixing continued at 100 RPM for 5 min. Formulations of the investigated OSM samples were as follows (Table 2). The samples were kept at constant temperature in dark conditions and were loaded into identical glass vessels. It should be noted that the glass microspheres (MS), even prior to the preparation of the OSM formulations, contained a fraction of broken microspheres. This was not the case for polymer microspheres (PM), which, in general, are far more durable during any kind of mechanical processing.

### 3.2. Density Measurement

A graduated cylinder (inner diameter = 22.45 mm) was used for the determination of OSM sample densities. Prior to each measurement, the cylinder was rinsed with nitric acid, then with deionised water and, finally, with HTP. The cylinder was then dried and weighed on a precision balance. Next, an OSM sample was loaded into the cylinder, following which the cylinder was placed on the precision balance, against a backdrop of millimeter paper, in a darkened chamber. The chamber was briefly opened once for every measurement, which consisted of recording the weight of the cylinder with the sample and the height of the OSM sample. These measurements were performed once every hour for the first 6 h since the OSM sample was produced, followed by measurements after 24 and 48 h had elapsed.

### 3.3. Time-Resolved Raman Spectroscopy

Raman spectroscopy was performed using a Raman microscope (inVia Renishaw, Wotton-under-Edge, UK), which was equipped with a CCD detector, using red (633 nm) laser excitation. Spectra were recorded in a static range of 100–1940 cm^−1^. All measurements were made in a backscattering geometry using a 50× microscope objective with a numerical aperture value of 0.75, providing scattering areas of 1 μm^2^. Single-point spectra were recorded with 4 cm^−1^ resolution and 10 s accumulation times.

**Figure A1:**
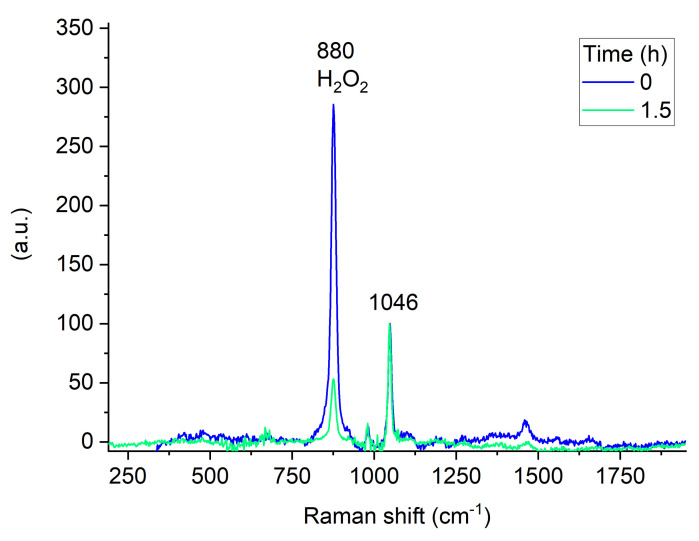
Raman spectra of OSM 1A in the beginning (blue line) and 90 min after preparing the material.

**Figure A2:**
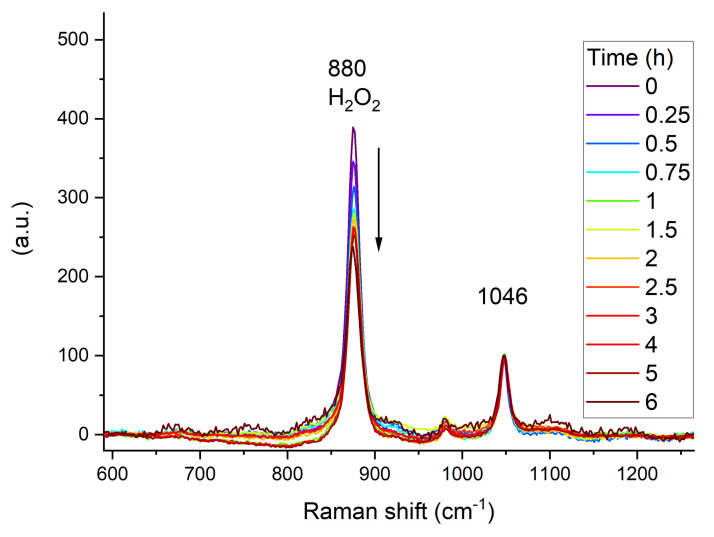
Raman kinetic spectra obtained by measurement of samples OSM 2A. Signal changes at 880 cm^−1^ symmetric vibration between O–O in hydrogen peroxide molecule as a function of time for tested OSM. The arrow indicates the direction of the signal changes).

The decomposition of hydrogen peroxide was observed in the OSM samples at constant temperature using Raman spectroscopy. The analytical signal peak at 880 cm^−1^ was used, corresponding to the symmetric vibration between O–O in the hydrogen peroxide molecule. The OSM samples were supplemented with 3.85 wt.% MgSO_4_, acting as an internal reference substance. All spectra were recorded in dark conditions, so as to prohibit any kind of photochemical reactions. The samples were homogenised and placed in the Raman spectrometer. The procedure of preparing the OSM samples and calibrating the spectrometer required approximately 10 min. Consequently, the first recorded Raman spectrum for each sample was listed at t = 10 min, with the following spectra being recorded after 6 h or 24 h had elapsed in total, depending on the observed dynamics of HTP decomposition in the samples (Figure A1 and Figure A2). Each spectrum was recorded as a single scan, in a fixed range of 100–1940 cm^−1^. One spectrum for each time point was acquired. The measurement results were standardised to the MgSO_4_ signal at 1046 cm^−1^ [39].

**Figure 4:**
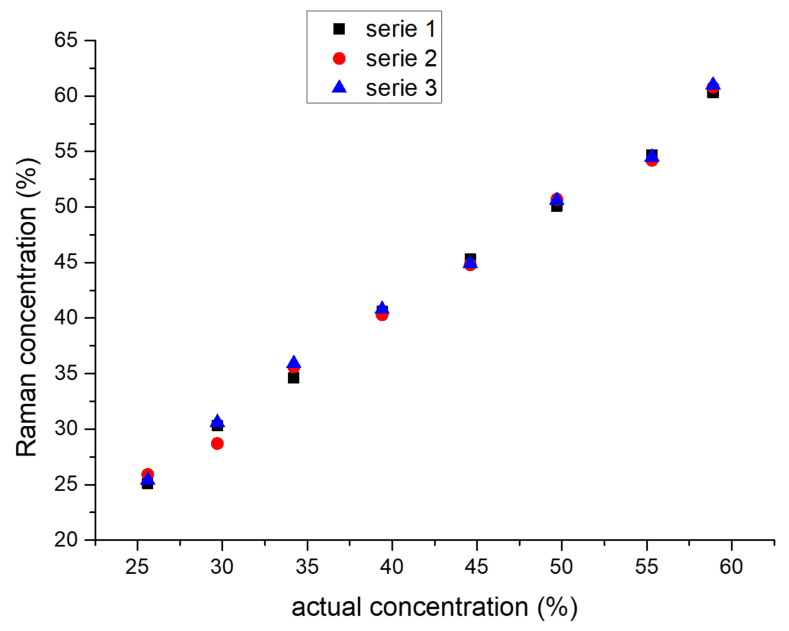
Calibration curve for the concentration of hydrogen peroxide in OSM samples validated via mangnaometric titration.

The use of a 60 wt.% aqueous solution of hydrogen peroxide (HTP) in the preparation of OSM explosive samples (Table 2) translates to a starting hydrogen peroxide concentration of 40.95 wt.% in OSM **1A**–**4D**. The calibration curve method was employed to corroborate the Raman signal with the concentration of hydrogen peroxide in aqueous solutions containing magnesium sulfate. The initially found concentrations were then calibrated to the actual concentration of hydrogen peroxide in OSM samples, based on the manganometric titration of such samples (Figure 4). A slight mismatch between the Raman signal for hydrogen peroxide solutions and the signal observed in OSM samples was found due to this additional calibration and was corrected in the results presented in this work.

## 4. Conclusions

The presented results evidence the significant impact of the choice of auxiliary oxidising agent, binder and even type of sensitising agent on the dynamics of the decomposition of hydrogen peroxide in OSM explosive formulations.

They key influence on the decomposition of hydrogen peroxide can be attributed to the choice of the auxiliary oxidising agent, as seen clearly by the comparison of the HTP decomposition in OSM 1A with that of virtually any other investigated sample. While a stark difference is seen in that case, significant differences are observed when the cation present in the auxiliary oxidising agent is exchanged (i.e., AN vs. SN vs. PN vs. CN), with OSM formulations utilising calcium nitrate (CN) showing the slowest HTP decomposition and being the only samples that maintain an effective concentration of hydrogen peroxide in excess of 30 wt.%, i.e., the threshold for maintaining the ability to undergo detonation, as indicated in the literature [27].

The choice of binder is also of significant importance, as not only does it affect the dynamics of HTP decomposition, but it also determines the stability and mechanical properties of the formulations. This is illustrated by the fact that utilising gum arabic as the binder (OSM **4D**) leads to phase separation in the samples, as well as their high fluidity. In contrast, the choice of HEC (OSM **4C**) results in a very viscous composition that maintains its shape even after stirring.

Glass microspheres are viewed as an inert sensitising agent for a variety of explosives, including emulsion explosives [40]. In the case of OSM formulations, this is not the case, as found in this work. Instead, glass microspheres noticeably increase the pace of HTP decomposition. This is evidenced by the increased pace of hydrogen peroxide decomposition for all samples utilising glass microspheres (MS), in comparison with samples utilising polymer microspheres (PM). This behaviour is likely due to fracturing of the MS that results in porous fragments, on which hydrogen peroxide may be adsorbed due to the fact that, in contact with water, silanol groups (-OH) are formed on the surface of the glass. At an approximately neutral pH, a significant part of the silanol groups is deprotonated, creating negative surface charges (-O^−^). The density of these negative charges is usually small and uncontrollable, but they can act as active catalytic sites, thereby facilitating the catalytic decomposition of hydrogen peroxide. The trace impurities present in the glass constituting the MS may also be a reason for the observed trend.

## Figures and Tables

**Table 1:** Velocity of detonation (VoD) of HTP-based EX formulations reported in the recent literature.

Fuels	Auxiliary Substances	VoD [m·s^−1^]	Ref.
Glycerine	ammonium nitrate, glass microspheres	5400–5700	[26]
Glycerine	glass microspheres/polymer microspheres/gas bubbles	3000–5500	[27]
Glycerine, Al	ammonium nitrate, glass microspheres	3600–5500	[28]
Glycerine	glass microspheres	2600–5100	[29]
Glycerine, Al	ammonium nitrate, glass microspheres	4400–5200	[30]

**Table 2:** Composition of samples investigated in this work, given in wt.%.

OSM: ^a^	1A	1B	1C	2A	2B	3A	3B	4A	4B	4C	4D
UltrAN	9.52	-	-	-	-	-	-	-	-	-	-
AN	-	9.52	9.52	-	-	-	-	-	-	-	-
SN	-	-	-	9.52	9.52	-	-	-	-	-	-
PN	-	-	-	-	-	9.52	9.52	-	-	-	-
CN	-	-	-	-	-	-	-	9.52	9.52	9.52	9.52
GG	2.91	2.91	2.91	2.91	2.91	2.91	2.91	2.91	-	-	-
LBG	-	-	-	-	-	-	-	-	2.91	-	-
HEC	-	-	-	-	-	-	-	-	-	2.91	-
GA	-	-	-	-	-	-	-	-	-	-	2.91
HTP	68.25										
Gl	14.52										
MS	-	-	0.95	-	0.95	-	0.95	0.95	0.95	0.95	0.95
PM	0.95	0.95	-	0.95	-	0.95	-	-	-	-	-
MgSO_4_	3.85	3.85	3.85	3.85	3.85	3.85	3.85	3.85	3.85	3.85	3.85

^a^ The composition of each OSM formulation (1A–4D) is the sum of the shares of components listed in its respective column. The components of the formulations have been grouped by type and colour-coded (auxiliary oxidising agents in green, binders in yellow, main oxidising agent and fuel in blue, sensitising agents in gray) for clarity.

**Table 3:** Materials used in this work.

Chemical (Code)	Purity Grade	Source
Ammonium nitrate porous prill (UltrAN) ^a^	>99.4%	Yara (Szczecin, Poland)
Ammonium nitrate (AN)	>95%	Nitrogen Plant “Pulawy” (Pulawy, Poland)
Sodium nitrate (SN)	>99%	POCH S.A (Gliwice, Poland)
Potassium nitrate (PN)	>99%	POCH S.A (Gliwice, Poland)
Calcium nitrate tetrahydrate (CN)	>99%	POCH S.A (Gliwice, Poland)
Hydrogen peroxide 60 wt.% solution (HTP)	analytical	EnvoLab Chemicals (Dlugomilowice, Poland)
Glycerine (Gl)	>99.5%	TechlandLab (Tarnobrzeg. Poland)
Guar gum S.C.-406 (GG)	>99%	Meyhall Chemical AG (Kreuzlingen, Germany)
Locust bean gum (LBG)	>99%	Sigma-Aldrich (Burlington, MA, USA)
Hydroxyethylcellulose (HEC)	>99%	Sigma-Aldrich (Burlington, MA, USA)
Gum arabic (GA)	>99%	Sigma-Aldrich (Burlington, MA, USA)
Glass microspheres type K-015 (MS)	n/a	3M (Saint Paul, MN, USA)
Polymer microspheres (PM)	n/a	AkzoNobel (Amsterdam, The Netherlands)
Magnesium sulfate (MgSO_4_)	>99%	POCH S.A (Gliwice, Poland)

^a^ Bulk density 670–720 kg/m^3^, prill diameter 1.0–2.0 mm.

## Data Availability

Data are available from the authors on request.

## Abbreviations

The following abbreviations are used in this manuscript:

AN	Ammonium nitrate
CCD	Charge-coupled device
CN	Calcium nitrate
EX	Explosives
GA	Gum arabic
GG	Guar gum
HEC	Hydroxyethylcellulose
HTP	Concentrated hydrogen peroxide
LBG	Locust bean gum
MS	Glass microspheres
OSM	On-site mixed
PM	Polymer microspheres
PN	Potassium nitrate
RPM	Rotations per minute
SN	Sodium nitrate
UltrAN	Ammonium nitrate porous prill
VoD	Velocity of detonation
